# Atlas of ACE2 gene expression reveals novel insights into transmission of SARS-CoV-2

**DOI:** 10.1016/j.heliyon.2020.e05850

**Published:** 2020-12-26

**Authors:** Kun Sun, Liuqi Gu, Li Ma, Yunfeng Duan

**Affiliations:** a Shenzhen Bay Laboratory, Shenzhen, 518132, China; b Department of Ecology and Evolutionary Biology, The University of Kansas, Lawrence, KS, 66045, USA; c Beijing Huayuan Academy of Biotechnology, Beijing, 100192, China

**Keywords:** COVID-19, 2019-nCov, Novel coronavirus, Potential host, Susceptibility

## Abstract

The recent pandemic, COVID-19, is caused by a novel coronavirus, SARS-CoV-2, with elusive origin. SARS-CoV-2 infects mammalian cells via ACE2, a transmembrane protein. Therefore, the conservation and expression patterns of ACE2 may provide valuable insights into tracing the carriers of SARS-CoV-2. In this work, we analyzed the conservation of ACE2 and its expression pattern among various mammalian species that are close to human beings. We show that mammalian ACE2 gene is deeply conserved at both DNA and peptide levels, suggesting that a broad range of mammals can potentially host SARS-CoV-2. We further report that ACE2 expression in certain human tissues are consistent with clinical symptoms of COVID-19 patients. Furthermore, we have built the first atlas of ACE2 expression in various common mammals, which shows that ACE2 expresses in mammalian tissues in a species-specific manner. Most notably, we observe exceptionally high expression of ACE2 in external body parts of cats and dogs, suggesting that these household pet animals could be vulnerable to viral infections and/or may serve as intermediate hosts, thus yielding novel insights into the transmission of SARS-CoV-2.

## 1 Introduction

Since December 2019, outbreak of COVID-19, a severe respiratory disease, has turned into a worldwide pandemic. After taking strong quarantine measures and national-wide lockdowns, the number of confirmed diagnoses is rapidly declining in China since February 2020. However, the endemic epicenter had since shifted to Italy and later to the United States. COVID-19 is becoming a global challenge to public health and continues to gather close attention. The culprit of this pandemic is a new virus named severe acute respiratory syndrome coronavirus 2 (SARS-CoV-2) [1], which belongs to the same beta coronavirus family as SARS-CoV and MERS-CoV, the other two viruses that caused outbreaks in the past two decades [1,2]. SARS-CoV-2 and SARS-CoV are closely related, and both invade human cells via attaching their S proteins to a host transmembrane protein called ACE2 (angiotensin converting enzyme 2) with several cofactors [2, 3, 4]. Using transcriptome data, previous studies reported that the receptor gene indeed expresses in the lungs [5,6]. The connections between ACE2 expression and viral infection are further supported by clinical cases from the United States, which confirmed the presence of SARS-CoV-2 in both the upper respiratory tract and stool sample of COVID-19 patients [7].

Some studies have suggested that the original host of SARS-CoV-2 may be bats [2,8]. However, in the case of COVID-19, the outbreak occurs in winter when bats are under hibernation, making them unlikely to be the direct source of human infection. Hence, SARS-CoV-2 is likely transmitted to humans through some (small) carnivores. Recent studies have pointed to pangolins and minks as other hosts of the virus [8, 9, 10]. In spite of popular efforts of virus tracing that is attracting much attention, is it possible that some of the animals living in close proximity to humans may also be susceptible to the virus and could potentially become additional hosts to SARS-CoV-2 hence further facilitating its transmission?

In attempt to address these questions, in this study, we focus on ACE2 gene, the host receptor of SARS-CoV-2. We evaluated the conservation of ACE2 genes across mammals and used quantitative data to infer their susceptibility to SARS-CoV-2 infection. In particular, our investigations were focused on species that live in close proximity with humans, i.e., pets and livestock. Our analyses identified potential species susceptible to SARS-CoV-2 and yielded novel insights into virus tracing and transmission, which may further contribute to the prevention and control of the COVID-19 pandemic.

## 2 Results

### 2.1 Conservation of ACE2 gene in mammals

**Figure 1:**
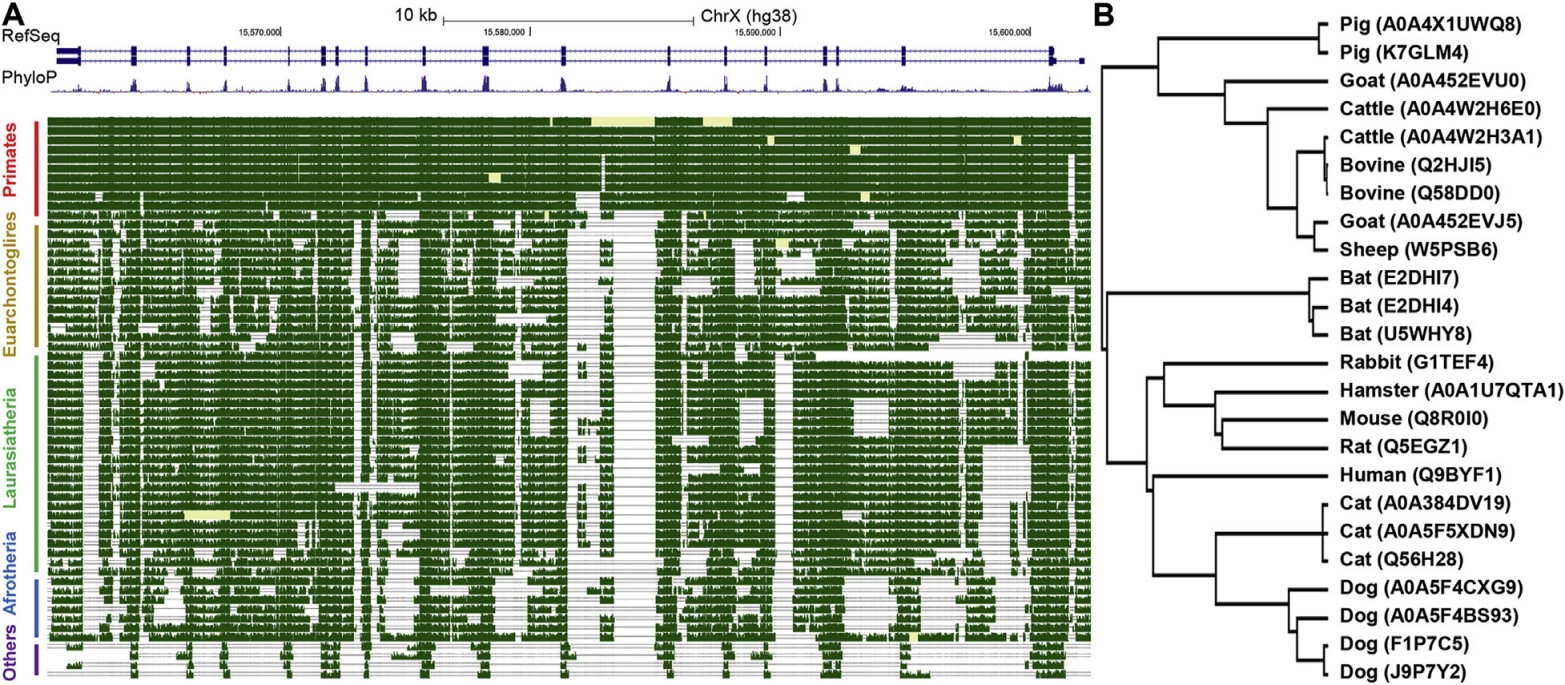
Conservation analysis of ACE2 gene. (A) Snapshot from the UCSC genome browser showing the DNA level conservation of ACE2 gene. Horse was removed due to lack of pair-wise alignment data. (B) Phylogenetic tree based on mammal ACE2 protein sequences from UniProt database. The top panel in (A) shows RefSeq annotation of ACE2 gene in human (which contains two isoforms, and the bars stand for exons), the middle panel shows conservation scores among 100 vertebrates, higher values denote higher levels of conservation, and the best-in-genome pairwise alignments among 42 mammals are plotted in the bottom panel. For (B), the protein accession number in UniProt are shown in parentheses and some species have multiple records.

**Figure 2:**
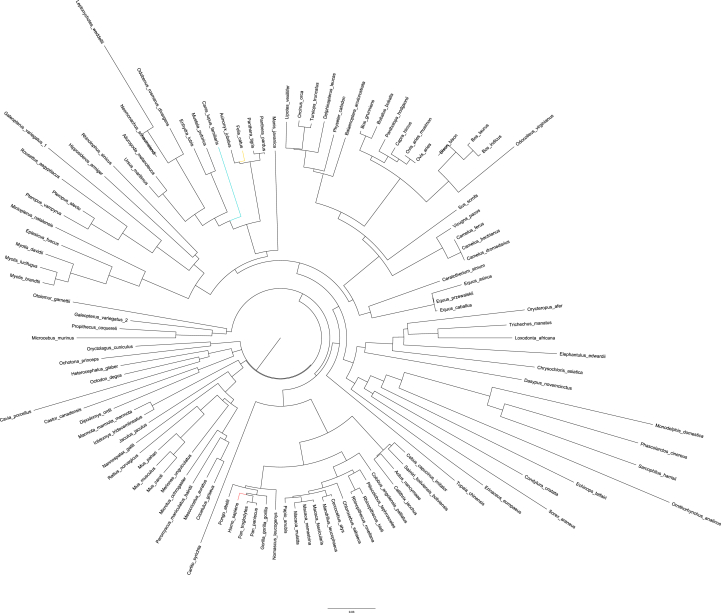
Phylogenetic tree based on mammal ACE2 protein sequences from OrthoDB database. Human, mouse, cat, and dog species are highlighted.

We first examined the conservation of ACE2 across mammalian species. We found that mammalian ACE2 genes are highly conserved at the DNA level (Figure 1A). To determine the conservation of ACE2 at the peptide level, we searched the UniProt database [11] for high-confidence ACE2 protein sequences in mammals. We limited the analysis to common mammalian species listed in Methods (plus rats) and discarded duplicate records as well as records with abnormally short sequences (<300 amino acids), leaving 24 ACE2 protein sequences for subsequent analyses. Pairwise alignment results showed that 23 non-human ACE2 protein sequences share high level similarity with the human counterpart, with a median identity score of 81.9% (range: 78.6%–85.2%). Notably, for the two virus-binding hotspots (i.e., the 31th and 353th amino acid in human ACE2 protein) [12], all the mammals except mice and rats share the same amino acids as the human ACE2 protein (Supplementary Table S1). Surprisingly, in contrast to conventional phylogenetic tree generated using genomic data, in the phylogenetic tree based on the ACE2 protein sequences, cats and dogs are the species clustered closest to humans instead of mice among the mammals included in this analysis (Figure 1B). In addition, a more comprehensive analysis using ACE2 homologues from more than 100 mammals in OrthoDB database [13] shows a similar result (Figure 2). Taken together, the conservation analysis shows that ACE2 gene is highly conserved among common mammals at both DNA and peptide levels, suggesting that SARS-CoV-2 can potentially bind to ACE2 proteins in these mammals (in particular, common domestic pets) with high affinity [2].

### 2.2 Expression profile of ACE2 gene in human tissues

**Figure 3:**
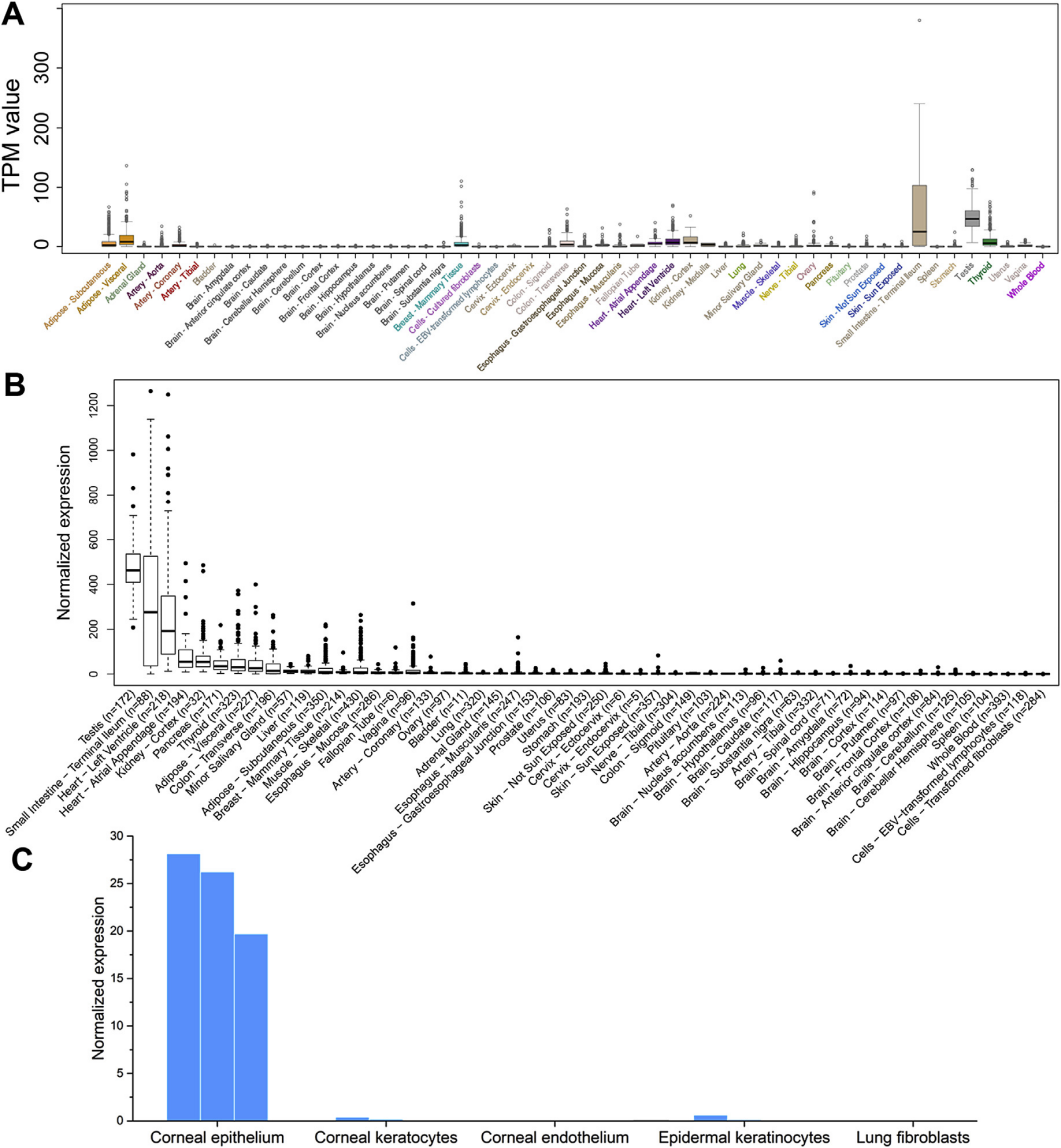
Expression of ACE2 gene in human tissues. (A) original TPM (Transcripts Per Kilobase of exon model per Million mapped reads) values, and (B) after normalization in GTEx project. (C) conjunctival and corneal tissues, epidermal keratinocytes, and lung fibroblasts. Available replicate experiments for tissues in panel C were shown in multiple bars.

We then profiled and normalized (see Methods) the expression patterns of ACE2 gene in human tissues. Data from the GTEx project show that ACE2 is expressed in various tissues, including testis, intestines, heart, kidney, and pancreas (Figure 3A and B). It is worth noting that our normalized ACE2 expression pattern is similar to the original version obtained from the GTEx portal, except that our analysis highlights the heart as the tissue with third highest ACE2 expression.

We also collected transcriptome data from tissues that are not in the GTEx datasets, including conjunctiva, cornea, epidermal keratinocytes, and lung fibroblasts, considering that these tissues are frequently exposed to the air. ACE2 gene is highly expressed in conjunctival and corneal epithelial cells (Figure 3C), suggesting that the eye could be vulnerable to SARS-CoV-2 infection. In contrast, ACE2 is not expressed in epidermal keratinocytes nor lung fibroblasts, which result is consistent with the GTEx data. The result is also consistent with a previous report based on single-cell RNA-seq data showing that ACE2 expression in lungs is limited to the alveolar type 2 (AT2) cells [14].

### 2.3 Expression pattern of ACE2 gene in mice

**Figure 4:**
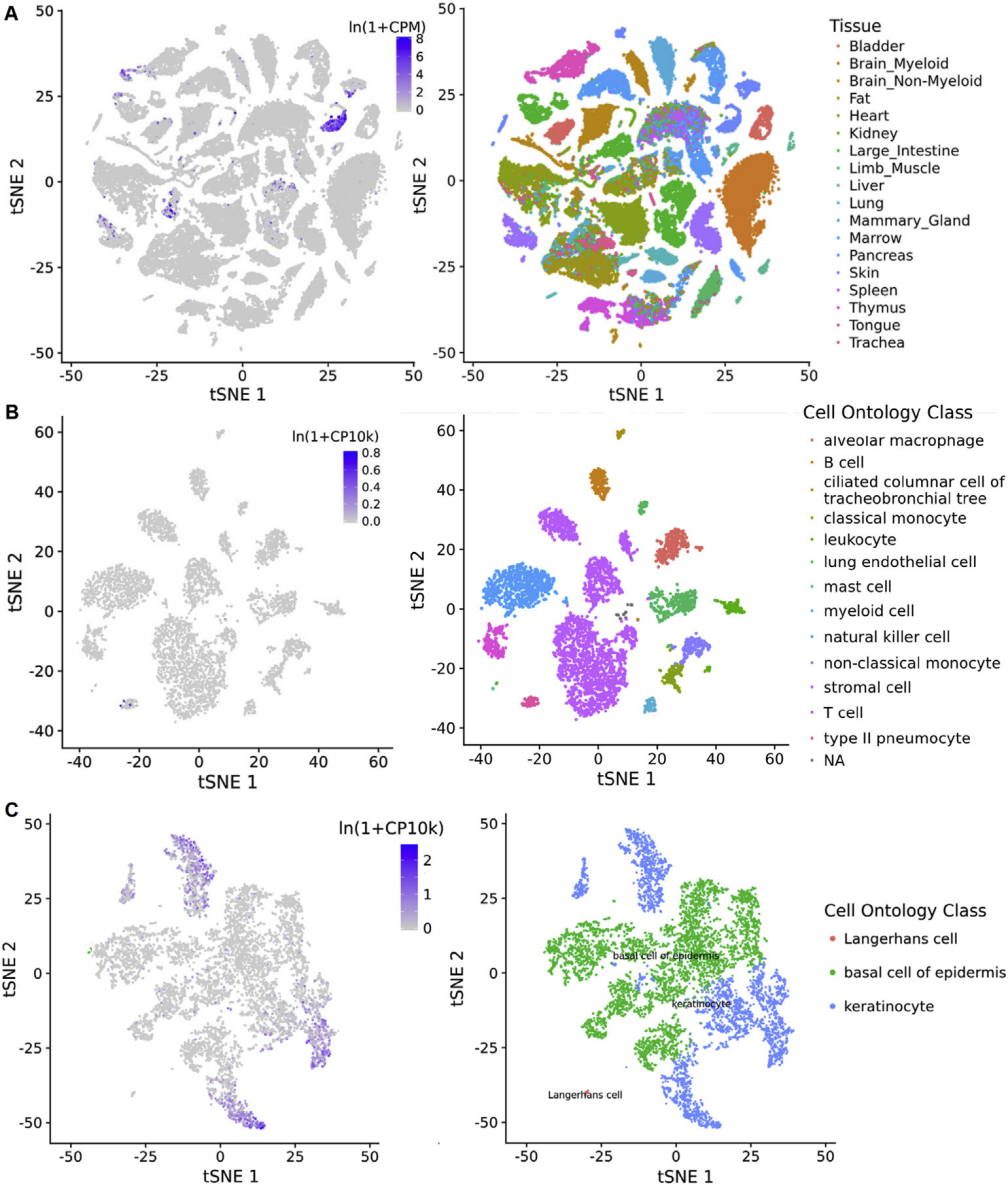
Expression of ACE2 in murine tissues. The data was obtained from Tabula Muris project (the FACS protocol subset). (A) Expression in all cells (left) and corresponding tissue origin of these cells (right). Note that the cells were clustered using t-SNE (t-distributed Stochastic Neighbor Embedding) algorithm based on their transcriptome. (B) Expression in lungs and (C) tongue.

Mouse is the most widely used model species in biomedical studies, including those related to SARS-CoV-2. We extracted the expression data of murine ACE2 gene from Tabula Muris project [15], which investigated various murine tissues using single-cell RNA-seq experiments. Murine ACE2 gene is expressed in kidney, heart, intestine, and pancreas, a pattern similar to human; however, murine ACE2 gene is not expressed in any cell types in lungs, while it expresses in tongue and skin (Figure 4). These observations suggest that ACE2 gene expression pattern could be species-specific among mammals. In particular, lung-related symptoms may not be expected when infecting normal mice with SARS-CoV-2.

### 2.4 Expression patterns of ACE2 gene in other mammals

**Figure 5:**
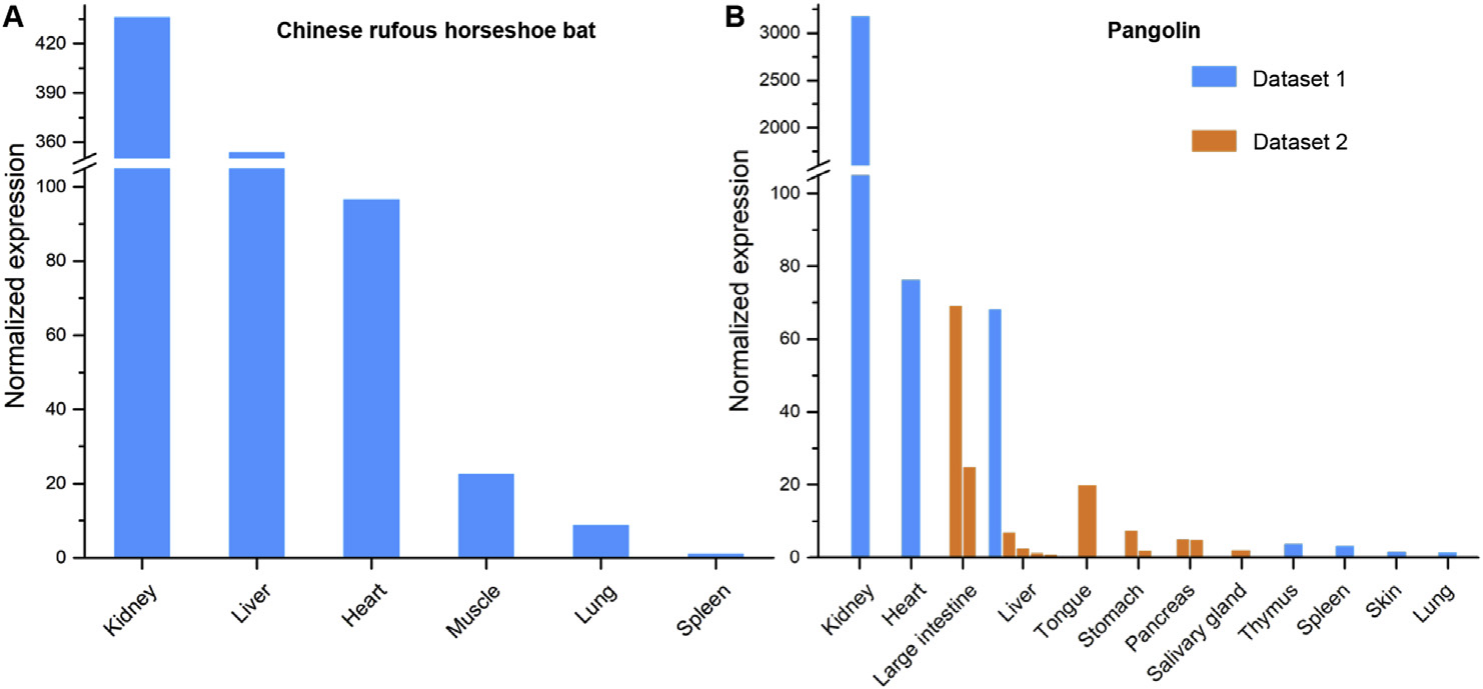
Expression of ACE2 gene in suspected intermediate host species. Expression levels in (A) bat, and (B) pangolin tissues. Two datasets for pangolin tissues were collected and labelled with different colors. Replicate experiments for some tissues were available and are shown as multiple bars.

Bats and pangolins are both hypothesized to be the natural hosts of SARS-CoV-2 [2,8]. We profiled the expression of ACE2 in various tissues in Chinese rufous horseshoe bats and Malayan pangolins. ACE2 gene is indeed expressed in most tissues examined, including those frequently exposed to the air, e.g., lungs in bats and tongue in pangolins (Figure 5).

**Figure 6:**
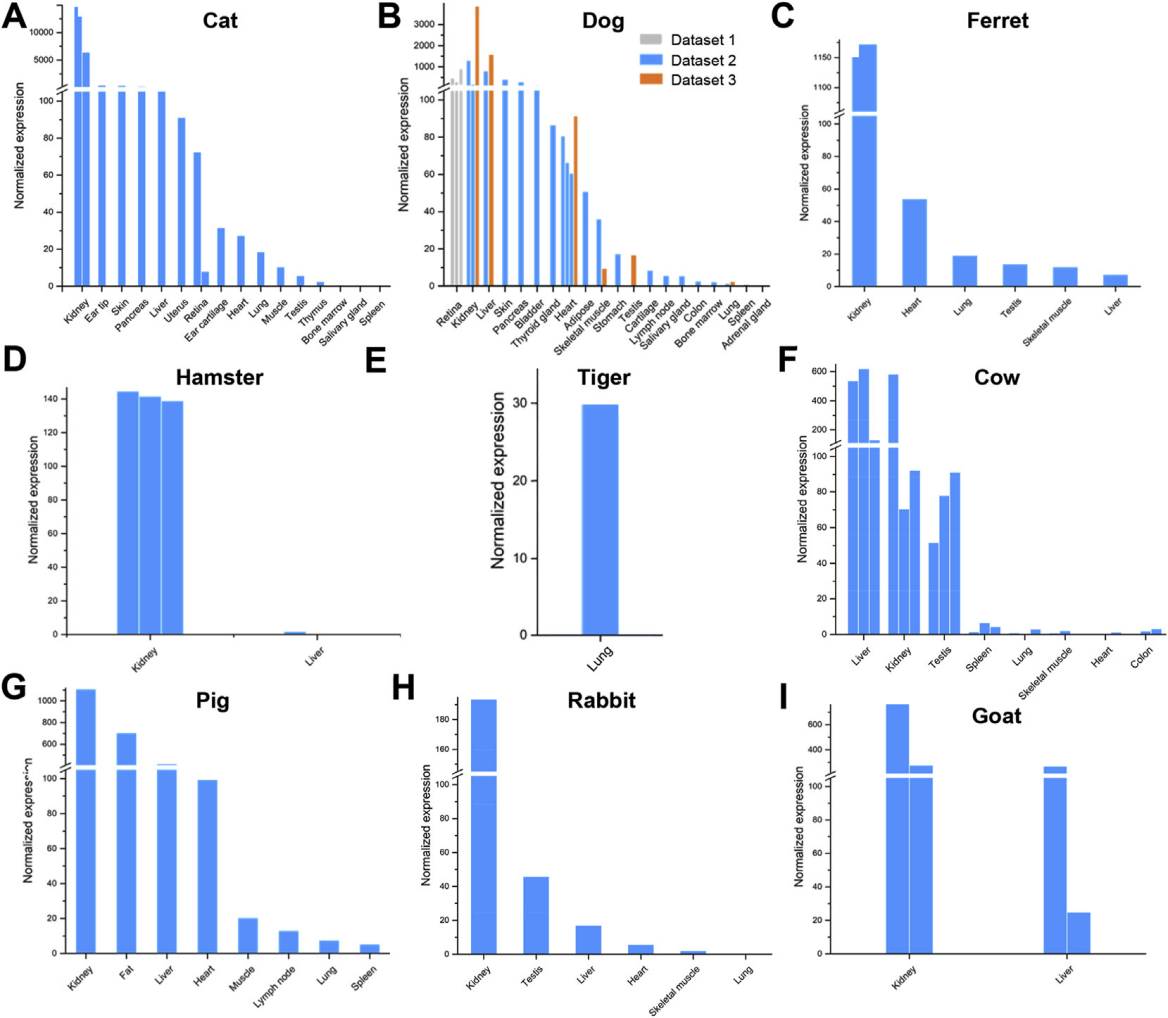
Expression of ACE2 gene in common pets and livestock. Expression levels in (A) cat, (B) dog, (C) ferret, (D) pig, (E) cow, (F) rabbit, (G) hamster, (H) goat, and (I) tiger tissues. Three datasets for dog tissues were collected and labelled with different colors. Replicate experiments for some tissues were available and are shown as multiple bars.

Pets are the most intimate animals to humans and may thus be contracted by human COVID-19 patients or to transmit the virus to humans if they are infected. We examined ACE2 expression patterns in cats and dogs, the most popular pets worldwide, as well as ferrets and hamsters, which are also common in China. ACE2 gene is highly expressed in various tissues in these animals, such as kidney, heart, and liver (Figure 6A–D). For cats, ACE2 is also highly expressed in skin, ear tip, lungs, and retina; for dogs, ACE2 is expressed in skin and retina. These observations suggest that cats and dogs may be highly susceptible to SARS-CoV-2 infection. In addition, we also observed ACE2 expression in the lungs of cats, ferrets and tigers (Figure 6E), which suggest that these animals may be more suitable for SARS-CoV-2 studies than rodent models [2,16].

Besides pets, livestock are another category of mammals that are frequently in direct contact with humans. We profiled the ACE2 expression in cows, pigs, rabbits (which can also serve as pets), and goats. In these animals, ACE2 is highly expressed in multiple tissues including kidney, liver, and heart, but not muscles (Figure 6F–I). Notably, ACE2 is also highly expressed in the fat of pigs. These results suggest that storage of fresh or undercooked meat, especially viscera tissues, may post potential risk of viral contaminations.

In addition, we also profiled the expressions of the known cofactors of ACE2, i.e., TMPRSS2, FURIN, and NRP1, which also play roles during the viral infection and are also conserved between human and the mammalian species investigated above. Briefly, these genes express much higher than ACE2 as well as more ubiquitously (Supplementary Table S2), suggesting that these cofactors may not become impediments during SARS-CoV-2 infection when ACE2 is present in certain cells.

## 3 Discussion

In this study, we investigated the conservation and profiled the expression patterns of ACE2 in various mammalian species. We show that the ACE2 expression profile in human tissues agrees with clinical observations of SARS-CoV-2 infected patients. For example, ACE2 is by far most strongly expressed in testis; SARS is known to cause orchitis and destruction to male germ cells [17] and SARS-CoV-2 infection is also supposed to impact the reproductive system [18]. Similarly, our analyses show that small intestine is the tissue with the second highest level of ACE2 expression, which is in line with reports showing that SARS-CoV-2 could be detected in stool samples of COVID-19 patients in China and other countries [7], even after the viral RNA has decreased to undetectable level in respiratory tract [19]. Additionally, our normalized data highlights the heart as the tissue with third highest ACE2 expression, which is in accord with clinical reports that COVID-19 patients complicated with cardiac diseases are subject to the highest mortality risk [20,21]. Our data also show that the eye is highly vulnerable and may serve as a route of virus infection, which is consistent with recent clinical reports [22]. Together, the consistence between our results and clinical observations demonstrates that ACE2 expression is a reasonable indicator for susceptibility to SARS-CoV-2 infection and tissue involvement in COVID-19.

In addition, we show that mammalian ACE2 genes are highly conserved across lineages and exhibit broad expressions. In particular, we had built the first comprehensive ACE2 expression atlas across 12 mammalian species and revealed species- and tissue-specific expression patterns of ACE2, which suggests certain mammals may be the hosts of SARS-CoV-2, i.e., carriers without significant symptoms. It is worth noting that mouse is a common model species for many human medical studies, including COVID-19; however, mice and rats are the only mammals that are different from human in ACE2 protein sequences at the two virus-binding hotspots, which is consistent with previous report that SARS-CoV-2 could not use murine ACE2 protein as a receptor to enter murine cells [2]. Moreover, ACE2 is barely expressed in all cell types in murine lungs. Thus, both observations argue against using mice as the optimal animal model for studying coronavirus related diseases.

Besides the species shown in Figure 1, a much broader range of species are also investigated in this (Figure 2) and others’ works [23,24]; however, we aim to focus on the animals that are in close proximity to human beings (e.g., pets and livestock) because we think that these species are more likely to serve as intermediate hosts of SARS-CoV-2 during the viral transmission thus deserve more attentions. Most notably, our analyses show that cats and dogs possess the most conserved ACE2 protein to human and they also highly express ACE2 gene in various tissues (Figure 1B, 6A and B). In cats, expression levels in top four ACE2 expression hotspot tissues are all magnitudes higher than any other mammals examined. Besides media reports on SARS-CoV-2 positive cats, dogs and even tigers, recent studies had confirmed that SARS-CoV-2 indeed could infect cats and spread among them [25,26]; another recent study had revealed human-to-dog transmission of the virus [27]. Our analysis and these studies are thus consistent and complementary to each other. Together, these facts suggest high possibility that cats and dogs can host SARS-CoV-2. Furthermore, high ACE2 expression levels in the exterior body parts of cats and dogs suggest that they could transmit the virus to others via skin-to-skin contact. Stray animals could be more serious transmitters of the coronavirus. It is estimated that there are approximately 500 million stray dogs and similar number of stray cats worldwide. Cats and dogs are sometimes slaughtered for meat, including a large proportion of stray ones. Indeed, SARS-CoV-2 positive stray cats had been reported [28] while currently there is no evidence that pets could transmit the virus to human. Hence, in effort to control the spread of SARS-CoV-2, people should pay more attention to protect their pets, e.g., prevent them from contacting COVID-19 patients and keep them away from stray animals, and never abandon them.

On the other hand, there are several limitations of the current study. Firstly, we had only surveyed the RNA expression of ACE2 without any protein-level data. This is largely because protein expression data in non-model species is largely lacking; nevertheless, recent studies had demonstrated that the protein and RNA levels of ACE2 gene expression are highly consistent among human tissues [29], suggesting that RNA expression data is still meaningful and reliable in inferring the gene expression pattern. For instance, Zhou and colleagues had confirmed the protein-level expression of ACE2 and its cofactors in human ocular surface [30], which data is consistent with our analysis (Figure 3B) and both studies suggest susceptibility to SARS-CoV-2 infection of the eye. Moreover, in human, there is an additional isoform of ACE2 expressing in some tissues (including the lungs), which encodes a truncated, dysfunctional ACE2 protein [31]. In the mammalian species analyzed in this study, however, we could not find any orthologs of the truncated isoform of ACE2, which may be attributed to the incompleteness of current gene annotations in these non-model species. In current mainstream RNA-seq protocols, the RNA transcripts/molecules are sonicated into small pieces to fit the second-generation sequencing machines, which makes it uneasy to differentiate the expression of the truncated ACE2 isoform from the functional one as they share quite a lot exons, thus may led to incorrect overestimation of ACE2 expression. Hence, protein-level expression data could be valuable to further confirm the findings from transcriptome analysis. In addition, even though high expression of ACE2 is essential for SARS-CoV-2 infection, tissues that highly express ACE2 do not necessarily mean high susceptibility to the infection, especially for epidermic tissue as its outer layer mostly comprise dead cells that prevent replication of the virus after infection. Hence, we think that our work should be considered as a resource of ACE2 expression in common mammals, which provides information for functional studies to further investigate the viral infections as well as clues towards discovery of the ultimate and intermediate hosts of SARS-CoV-2 during the viral transmission.

## 4 Materials and methods

### 4.1 Reference genomes and gene annotations

A total of 13 mammalian species were investigated in this study (Table 1): *Homo sapiens* (human), *Mus musculus* (mouse), *Rhinolophus sinicus* (Chinese rufous horseshoe bat), *Manis javanica* (pangolin), *Felis catus* (cat), *Canis lupus familiaris* (dog), *Mustela putorius furo* (ferret), *Mesocricetus auratus* (hamster), *Bos taurus* (cow), *Sus scrofa* (pig), *Oryctolagus cuniculus* (rabbit), *Capra hircus* (goat), and *Panthera tigris* (tiger). Latest versions of reference genomes and RefSeq gene annotations [32] for these species were downloaded from National Center for Biotechnology Information [33].

**Table 1:** Reference genomes and gene annotations used in this study.

Species	Common name	Assembly name	Assembly accession	Annotation release ID
*Homo sapiens*	Human	GRCh38.p13	GCF_000001405.39	109
*Rhinolophus sinicus*	Bat	ASM188883v1	GCF_001888835.1	100
*Manis javanica*	Pangolin	ManJav1.0	GCF_001685135.1	100
*Felis catus*	Cat	Felis_catus_9.0	GCF_000181335.3	104
*Canis lupus familiaris*	Dog	CanFam3.1	GCF_000002285.3	105
*Mustela putoriu*s *furo*	Ferret	MusPutFur1.0	GCF_000215625.1	101
*Mesocricetus auratus*	Hamster	MesAur1.0	GCF_000349665.1	102
*Bos Taurus*	Cow	ARS-UCD1.2	GCF_002263795.1	106
*Sus scrofa*	Pig	Sscrofa11.1	GCF_000003025.6	106
*Oryctolagus cuniculus*	Rabbit	OryCun2.0	GCF_000003625.3	102
*Capra hircus*	Goat	ARS1	GCF_001704415.1	102
*Panthera tigris*	Tiger	PanTig1.0	GCF_000464555.1	101

### 4.2 Transcriptome data collection

Transcriptome data from 191 RNA-seq experiments (whole transcriptome shotgun sequencing) was collected from publicly available sources. Briefly, human data was from the GTEx (Genotype-Tissue Expression) project [34], ENCODE (ENCyclopedia Of DNA Elements) project [35,36], and [37]; bat data was from [38]; pangolin data was from [39] and [40]; cat data was from 99 Lives Cat Genome Sequencing Initiative project; dog data was from [41,42] and [43]; ferret and rabbit data was from [44]; hamster and goat data was from [45]; cow data was from [46]; pig data was from [47]; and tiger data was from [48]. Detailed information on data sources, including the accession numbers and tissues for each species, was listed in Supplementary Table S2.

### 4.3 Transcriptome data analysis

All the transcriptome data was analyzed using a unified pipeline. Briefly, raw RNA-seq reads were first preprocessed to trim sequencing adapters and low-quality cycles using *Ktrim* software [49] with default parameters. The preprocessed reads were then aligned to corresponding reference genomes using *STAR* software [50] with default parameters. Key statistics during preprocessing and alignment is presented in Supplementary Table S2. The vast majority (99.3%) of the samples have more than 10 million uniquely mapped reads, indicating sufficient sequencing depths for reliable gene expression quantifications [51], which was performed using *featureCounts* [52] software with default parameters against RefSeq gene annotations [32]. Considering that the reference genomes and gene annotations for most of the species included in this study are far from complete, we used ACTB (Actin Beta) gene from each RNA-seq experiment to normalize ACE2 expression for appropriate comparisons across species and tissue types instead of directly utilizing the conventional FPKM (Fragments Per Kilobase of transcript per Million mapped reads) values. ACTB is a housekeeping gene that is abundantly and stably expressed in most cell types, and is commonly used as an internal control for gene expression normalizations [53]. Importantly, ACTB gene is also conserved in all the species investigated in this study. The following formula was used to normalize ACE2 expression:

$$ACE2_{norm1}=\frac{No.\;of\;reads\;mapped\;to\;ACE2\;/\;length\;of\;ACE2}{No.\;of\;reads\;mapped\;to\;ACTB\;/\;length\;of\;ACTB}\times 10000$$

We used 10000 as the scaling factor here because ACE2 is typically expressed at much lower levels than ACTB. In addition, 2723 human housekeeping genes [54] are conserved in all the mammalian species investigated; we thus used the mean expression of these genes as additional normalization factor using the following formula:

$$ACE2_{norm2}=\frac{No.\;of\;reads\;mapped\;to\;ACE2\;/\;length\;of\;ACE2}{mean\left(conserverd\;housekeeping\;gene\;/\;length\;of\;that\;gene\right)}\times 10000$$

**Figure 7:**
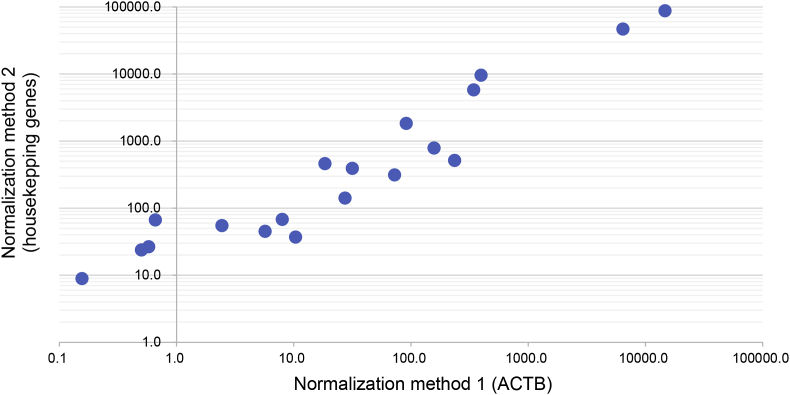
Correlation of the two normalization methods on the Cat dataset.

We found that the normalized expression values using these two approaches were highly consistent (Figure 7), therefore we used the results from the first approach to generate figures present in this work, and the results from the second approach could be found in Supplementary Table S2. Such normalization approaches were also applied to TMPRSS2, FURIN, and NRP1, the known cofactors of ACE2. Meanwhile, since human data from the GTEx (Genotype-Tissue Expression) project [34] was provided as preprocessed values, these values were used directly in the above formula in lieu of number of mapped reads and gene lengths.

## Declarations

### Author contribution statement

K. Sun: Conceived and designed the experiments; Performed the experiments; Wrote the paper.

L. Gu: Performed the experiments; Wrote the paper.

M. Ma: Performed the experiments; Analyzed and interpreted the data.

Y. Duan: Conceived and designed the experiments; Wrote the paper.

### Funding statement

This work was supported by Shenzhen Bay Laboratory and Beijing Huayuan Academy of Biotechnology.

### Data availability statement

Data associated with this study has been deposited at Gene Expression Omnibus (https://www.ncbi.nlm.nih.gov/geo) under the accession number GSE121922, GSE135455, GSE97638, GSE97638, GSE106077, GSE43013, GSE41637, GSE106077, and GSE43013; Sequence Read Archive (https://www.ncbi.nlm.nih.gov/sra) under the accession codes: SRP063381, SRP156258, SRP064341, SRP071078, SRP114662, ERP009821, and SRP032170.

### Declaration of interests statement

The authors declare no conflict of interest.

### Additional information

No additional information is available for this paper.
