# Supplementary material for: Atlas of ACE2 gene expression reveals novel insights into transmission of SARS-CoV-2
Source: Heliyon. 2020 Dec 26;7(1):e05850. doi: 10.1016/j.heliyon.2020.e05850 (PMC7762714; doi:10.1016/j.heliyon.2020.e05850)
Supplement: Supplementary.materials.20201220 [file mmc1.docx]

**Atlas of ACE2 gene expression reveals novel insights into transmission of SARS-CoV-2**

Kun Sun, Liuqi Gu, Li Ma, Yunfeng Duan

**Supplementary tables**

**Table S1.** Alignment result of mammal ACE2 protein sequences in UniProt database.

**Table S2.** Accession and statistics of the transcriptome data compiled in this study, as well as the normalized expression values of ACE2 and its known cofactors.
